# A national cohort study on hemodialysis arteriovenous fistulas after kidney transplantation - long-term patency, use and complications

**DOI:** 10.1186/s12882-021-02550-4

**Published:** 2021-10-19

**Authors:** Barbara Vajdič Trampuž, Miha Arnol, Jakob Gubenšek, Rafael Ponikvar, Jadranka Buturović Ponikvar

**Affiliations:** 1 Department of Nephrology, University Medical Centre Ljubljana, Ljubljana, Slovenia; 2 Faculty of Medicine, University of Ljubljana, Zaloška 7, 1000 Ljubljana, Slovenia

**Keywords:** Dialysis arteriovenous fistula, Kidney transplantation, AVF survival, AVF complications, AVF thrombophlebitis, AVF related surgery

## Abstract

**Objective:**

To describe the long-term hemodialysis arteriovenous fistula (AVF) patency, incidence of AVF use, incidence and nature of AVF complications and surgery in patients after kidney transplantation.

**Patients and methods:**

We retrospectively analysed the AVF outcome and complications in all adult kidney allograft recipients transplanted between January 1st, 2000 and December 31, 2015 with a functional AVF at the time of transplantation. Follow-up was until December 31, 2019.

**Results:**

We included 626 patients. Median AVF follow-up was 4.9 years. One month after kidney transplantation estimated AVF patency rate was 90%, at 1 year it was 82%, at 3 years it was 70% and at 5 years it was 61%; median estimated AVF patency was 7.9 years. The main cause of AVF failure was spontaneous thrombosis occurring in 76% of AVF failure cases, whereas 24% of AVFs were ligated or extirpated. In a Cox multivariate model female sex and grafts were independently associated with more frequent AVF thrombosis. AVF was used in about one third of our patients. AVF-related complications occurred in 29% of patients and included: growing aneurysms, complicated thrombosis, high-flow AVF, signs of distal hypoperfusion, venous hypertension, trauma of the AVF arm, or pain in the AVF/arm.

**Conclusions:**

AVFs remain functional after kidney transplantation in the majority of patients and are often re-used after graft failure. AVF-related complications are common and require proper care.

## Introduction

An arteriovenous fistula (AVF) is the best vascular access for hemodialysis patients [1, 2]. After successful kidney transplantation, it remains unused, but functional in many patients. There are no guidelines or generally accepted policies for the management (preservation or closure) of AVFs after kidney transplantation [3, 4]. The UK Renal Association vascular access guidelines does not provide guidance on the management of AVF after kidney transplantation [5]. The European Best Practice Guideline on Vascular Access mentions a possible improvement in cardiac function after AVF ligation, but does not recommend routine ligation after kidney transplantation [6]. The new National Kidney Foundation vascular access guidelines represent a fresh approach to vascular access care by emphasizing a more patient-focused approach and recommend development of the end-stage kidney disease Life Plan when choosing vascular access and planning up front also for transplant patients with failing grafts [7]. At a transplant unit, the presence of patent, but unused AVF is sometimes overlooked and questions about long-term patency and the incidence of AVF complications or systemic fistula effects remain unanswered. On the other hand, can a functional AVF be of any practical value even after successful transplantation? Some transplanted patients have a poor quality of peripheral veins or vascular access problems at the time of transplantation or thereafter. We are relieved when such a patient has a patent AVF that can be used for administration of intravenous therapy, dialysis, or therapeutic plasma exchange, especially in urgent cases.

There is scarce information in literature regarding the long-term patency of AVFs and their potential use after kidney transplantation. Moreover, literature on AVF complications after kidney transplantation is even scarcer. In our previously published cohort, complications with AVFs occurred in 12.5% of kidney transplant recipients, excluding asymptomatic or minimally symptomatic thrombosis and aneurysms [8].

To fill this gap in knowledge, we aimed to evaluate, in this retrospective observational cohort study, long-term AVF patency, incidence of its use and complications, and AVF-related surgery after kidney transplantation. This study adds relevant information on the long-term outcomes of hemodialysis access conduits in patients that do not utilize their access but might need it in the future.

## Patients and methods

Our retrospective observational cohort study was conducted at the University Medical Centre.

Ljubljana (Centre for Kidney Transplantation, Department of Nephrology), where all adult Slovenian patients with end-stage kidney disease are transplanted and monitored until graft failure and for one year thereafter. The study was approved by the Slovenian National Medical Ethics Committee (Approval No. 44/09/14). Due to the Slovenian National Medical Ethics Committe the informed consent was not required, because the data collection was part of the routine clinical procedure and was managed by the therapeutic team for purposes of improving patient management.

We retrospectively analysed the medical records of all adult kidney allograft recipients transplanted between January 1st, 2000 and December 31, 2015, who had a functional AVF at the time of transplantation. The observation period for each patient/AVF began at the time of transplantation, which was defined as baseline. Each AVF was followed until December 31, 2019, AVF thrombosis/ligation/extirpation, kidney graft failure, or recipient death, whichever occurred first. The collected data included demographic data (age at time of transplantation, sex, and underlying cause of end-stage kidney disease) and specific information about AVF: anastomosis site, type of AVF (native/polytetrafluoroethylene (PTFE)), AVF patency duration, cause of AVF dysfunction, incidence of AVF complications (thrombosis, thrombosis with thrombophlebitis, growing aneurysms, high flow, steal syndrome, venous hypertension with arm edema, trauma with bleeding), and treatment of complications (medical or surgery).

AVF data were collected during regular visits to the outpatient transplant clinic by a nephrologist or a fellow examining the patient. Clinic visits were scheduled weekly in the first month after transplantation, every other week in the second month, monthly until the end of the first year, and every three months thereafter. The functional status of the AVF was examined at each visit. In case of clinical abnormalities or complaints, a more detailed examination of vascular access was performed by the vascular access specialist, including: clinical assessment of aneurysms, skin quality and risk of rupture, inflammation, arm swelling, and clinical signs of steal syndrome. A complication of AVF was diagnosed for the purpose of this study, if a patient was referred to a vascular access specialist from the transplant clinic for a suspicion of a complication and the complication was confirmed by the vascular access specialist, usually on the grounds of clinical examination.

The primary objective of the study was to describe long-term AVF patency after transplantation. Secondary objectives included incidence of AVF use, incidence and type of AVF complications with emphasis on thrombosis with thrombophlebitis, identification of possible predictive factors of AVF thrombosis, and incidence of AVF-related surgery.

### Statistical analysis

Continuous variables with normal distribution were expressed as mean ± standard deviation. Categorical variables were reported as frequencies and percentages. Continuous variables were compared between groups using the Student’s t-test. The Chi-square test was used for comparing categorical variables. Kaplan-Meier survival analysis was used to present data on kidney graft survival (censored for patient death) and AVF patency (censored for kidney graft failure and patient death). To analyse factors affecting AVF thrombosis (excluding patients with AVF ligature), we have used Cox proportional hazards model. All statistical analyses were performed using Statistica 12.0 (StatSoft Europe, Hamburg, Germany).

## Results

### Study population

**Fig. 1:**
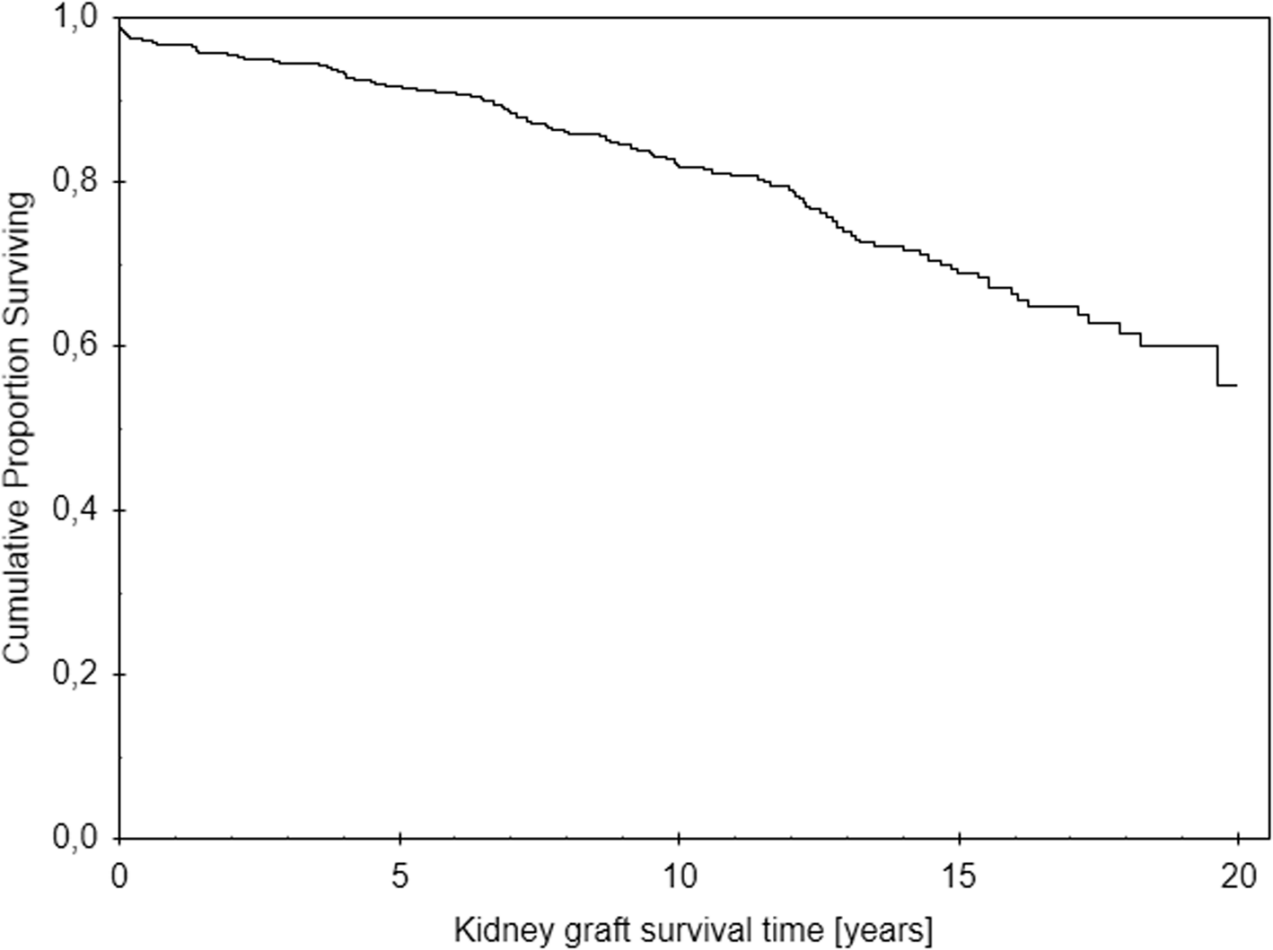
Kaplan-Meier survival curve, estimating kidney graft survival in our cohort of patients with a functional arterio-venous fistula at kidney transplantation

Between January 1st, 2000 and December 31, 2015, (altogether) 757 adult patients received a kidney allograft at the University Medical Centre Ljubljana. Among these, 626 (82.7%) had a.

functioning AVF at the time of transplantation and were enrolled in the study. Their baseline demographic and clinical characteristics are shown in Table 1. The majority of patients had a typical forearm radiocephalic AVF, while others had native AVFs or PTFE grafts located in the upper arm or a PTFE graft in the thigh. Standard immunosuppressive protocol included an induction with anti-interleukin-2 monoclonal antibodies and triple immunosuppressive maintenance therapy with calcineurin inhibitor, corticosteroids and an antimetabolite. The Kaplan-Meier estimated kidney graft survival of our cohort is shown in Fig. 1; 1-year estimated kidney graft survival was 97%, 5-year 92% and 10-year 82%.

**Table 1:** Baseline demographic and clinical characteristics of the study population^*^

Baseline characteristics	Value
N	626
Age at transplant (years)	48 ± 11 (18 to 77)
Recipients older than 65 years	40 (6.3%)
Male gender	380 (60.7%)
Cause of end-stage kidney disease:	
• Glomerulonephritis	196 (31.3%)
• ADPKD	97 (15.5%)
• Diabetes mellitus	47 (7.5%)
• (DM type 1)	23
• (DM type 2)	24
• Arterial hypertension	45 (7.2%)
• Vesicoureteral reflux	26 (4.1%)
• Pyelonephritis	25 (4%)
• FSGS	18 (2.9%)
• Alport syndrome	16 (2.5%)
• Other	156 (25%)
Graft type:	
• deceased donor	624 (99.7%)
• living related donor	2 (0.3%)
Concurrent other organ transplantation	14 (2.2%)
Time from AVF construction to transplant (months)	66 ± 49 (2 to 326)
AVF site	
• forearm	521 (83.2%)
• upper arm	105 (16.8%)
AVF nature	
• native	605 (96.7%)
• PTFE graft	21 (3.3%)
Delayed graft function	145 (23.1%)

Data are presented as total numbers (percentages) or mean ± standard deviation (range)

*Abbrevitions*: *ESKD* end stage kidney disease, *ADPKD* autosomal dominant polycystic kidney disease, *FSGS* focal segmental glomerulosclerosis, *AVF* arteriovenous fistula, *PTFE* polytetrafluoroethylene

### Long-term AVF patency

**Fig. 2:**
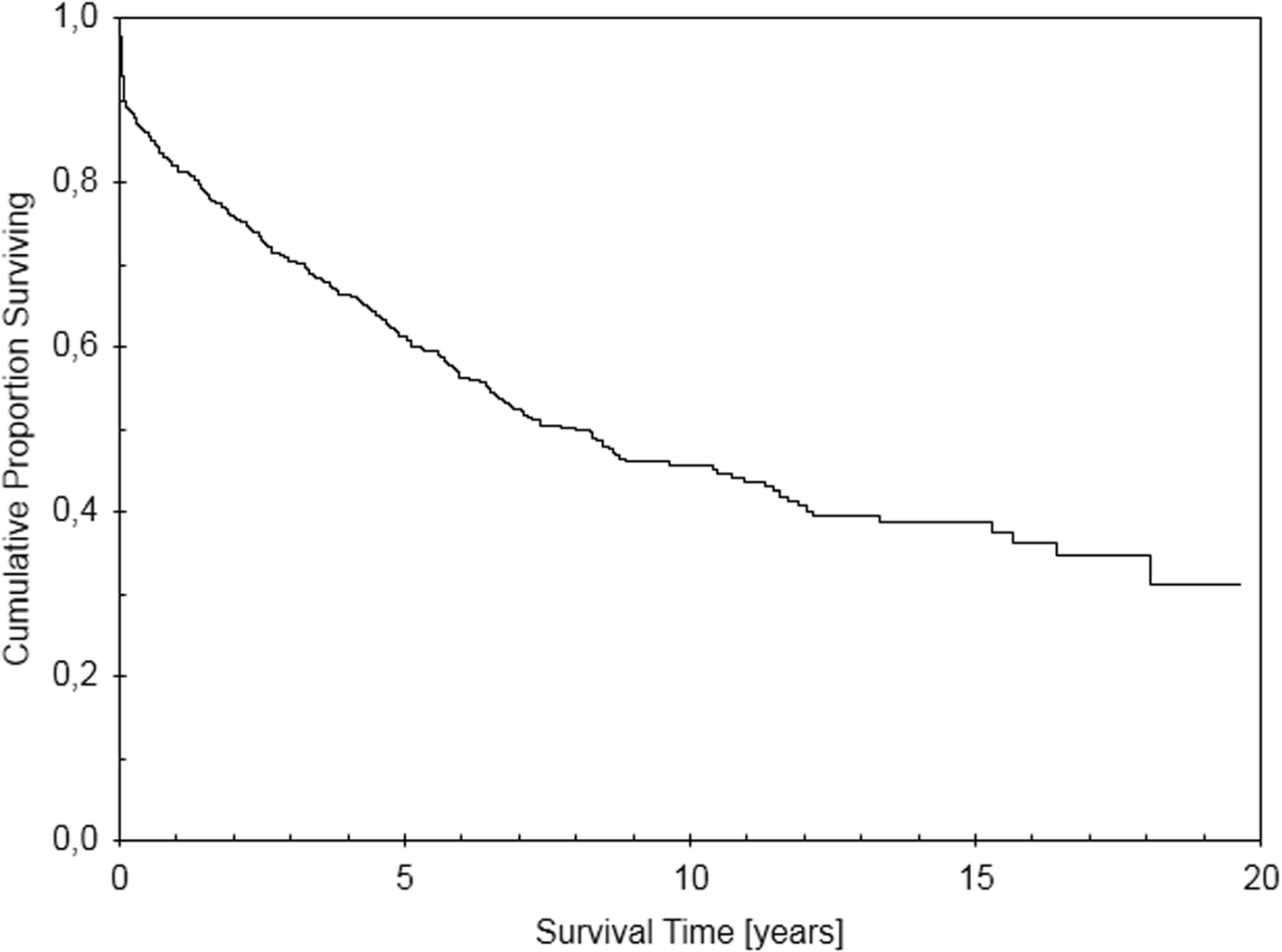
Kaplan-Meier survival curve, estimating AVF survival/patency after kidney transplantation; data are censored for kidney graft failure and patient death

Long-term patency of 603 patients with available AVF data is presented in Fig. 2. Median AVF follow-up was 4.9 years. One month after kidney transplantation estimated AVF patency rate was 90%, at 1 year it was 82%, at 3 years it was 70% and at 5 years it was 61%; median estimated AVF patency was 7.9 years. The main cause of AVF failure was spontaneous thrombosis occurring in 76% of AVF failure cases, whereas 24% of AVFs were ligated or extirpated. For AVFs that spontaneously thrombosed, we aimed to identify the baseline factors associated with thrombosis of AVF after kidney transplantation. In a Cox multivariate model male sex was independently associated with less frequent and grafts with more frequent AVF thrombosis (see Table 2).

**Table 2:** Cox proportional hazards model of baseline factors, affecting spontaneous AVF thrombosis after kidney transplantation. Data are presented as hazard ratios (HR) with 95% confidence intervals (CI) and *P* values

Baseline characteristic	HR (95% CI)	***P*** value
Age (years)	1.00 (0.99 - 1.01)	0.83
Male gender	0.63 (0.48 - 0.82)	< 0.001
Time from AVF construction to transplantation (months)	1.00 (1.00 - 1.00)	0.27
AVF site (proximal vs. distal)	1.01 (0.69 - 1.47)	0.97
AVF type (graft vs. native)	3.13 (1.81 - 5.43)	< 0.001

### AVF use after kidney transplantation

After kidney transplantation, AVF was used in about one third of our patients with a functioning AVF (see Table 3). In the majority of patients, AVF was used as a vascular access for hemodialysis (in case of delayed graft function or graft failure). In others, AVF was used as a vascular access for therapeutic plasma exchange (in case of antibody-mediated rejection or recurrent focal segmental glomerulosclerosis); the existing AVF being functional, the placement of a central venous catheter was not necessary. Furthermore, in many of these patients AVF was also used as a vascular access for intravenous therapy (i.e., antibiotics, immunosuppressants, blood products administered during or after hemodialysis or therapeutic plasma exchange).

**Table 3:** Reasons for AVF use after kidney transplantation. The categories are not mutually exclusive, i.e. there could be more than one reason for AVF use in a single patient

Extracorporeal procedure	N of patients (%)
Hemodialysis	
for delayed graft function	150 (23.9%)
for graft failure	53 (8.4%)
Therapeutic plasma exchange	
for antibody-mediated rejection	27 (4.3%)
for recurrent focal segmental glomerulosclerosis	6 (0.9%)

During follow-up, 132 patients (20.7%) experienced graft failure. Among these, 127 started hemodialysis, 53 (40.1%) with their original pre-transplant AVF and 12 (9.1%) with a newly constructed or reconstructed original AVF, while 62 (47%) patients started hemodialysis with a central venous catheter. Among the 74 patients who started dialysis with a catheter or newly constructed AVF, the original AVF was ligated/extirpated in 7 (9.4%) of them during the transplant period. In addition, 3 patients started peritoneal dialysis, while no information on vascular access or dialysis modality was available for two patients.

### AVF-related complications and surgery

AVF-related complications, requiring specialist consultation or surgery, occurred in 183/626 patients (29.2%) and included: growing aneurysms, complicated thrombosis (i.e., thrombosis with thrombophlebitis, central vein thrombosis or thrombosis/embolism of the feeding artery), occurrence of high-flow AVF, signs of distal hypoperfusion, venous hypertension with arm edema, trauma of the AVF arm, or pain in the AVF/arm (see Table 4). Surgical treatment of AVF-related complications was performed in 97 of our patients (15.5%). Surgery was required in 33 patients for aneurysms (27 extirpations, 5 aneurysmorraphies, 1 ligation), in 27 patients for thrombosed AVF (24 extirpations, 3 thrombectomies with reanastomosis), in 23 patients for high-flow fistulas (12 ligations, 7 bandings, 4 extirpations), for steal syndrome in 7 patients (all ligations), for venous hypertension and arm swelling in 5 patients (4 ligations, 1 extirpation), and for trauma or pain in 2 patients (2 extirpations). The operations were performed either by nephrologists dedicated to vascular access management under local anesthesia (ligation, banding, thrombectomy with reanastomosis, aneurysmorraphy), or by vascular surgeons under general anesthesia (extirpation).

**Table 4:** AVF-related complications and surgery after kidney transplantation

Type of AVF-related complication	Patients with complications, referred to a vascular access specialist^a^	Patients with complications requiring surgery^b^
Growing aneurysms^c^	84 (46%)	33 (39%)
Complicated thrombosis	53 (29%)	27 (51%)
• with thrombophlebitis • central vein / artery involvement		
High-flow AVF^d^	29 (16%)	23 (79%)
Distal hypoperfusion	7 (4%)	7 (100%)
Venous hypertension with arm edema	7 (4%)	5 (71%)
Trauma/Pain	3 (2%)	2 (66%)

^a^Data are presented as frequency (percentage - of all patients referred due to a complication)

^b^Data on surgical procedures are presented as frequency (percentage - of patients with a complication needing surgical treatment)

^c^i.e. aneurysms growing in size as detected by the patient; surgery was generally performed if aneurysms were of sufficient size to present an aesthetic or safety problem

^d^i.e. a patient referred to a vascular access specialist due to suspected high-flow AVF; there was no exact definition of high-flow AVF and flow measurement was not performed in all patients; a flowreduction or ligation was generally performed if there were negative consequences on the cardiovascular system (significant heart failure or pulmonary hypertension) or if the kidney graft function was good and there was an alternative option for future vascular access

### AVF-related thrombophlebitis

Of the 235 AVFs that thrombosed, 50 (21%) had thrombosis complicated with significant thrombophlebitis occurring 12 to 144 months after transplantation; other thromboses were asymptomatic. Among the 50 cases of AVF thrombosis with thrombophlebitis, 64% of AVFs were located in the forearm, 34% in the upper arm, and 2% in the thigh. In the majority (66%) of cases, the thrombosed AVF had aneurysms. Clinical presentation included redness, tenderness, and swelling along the course of the fistula vein in all patients; four patients (8%) were also febrile. CRP was measured in 20 patients (40%) with severe thrombophlebitis: the mean CRP at the time of presentation was 60 (range: 3 to 169) mg/L. Deep vein thrombosis was documented in one patient (2% of patients with thrombophlebitis and 0.4% of all patients with thrombosed AVF) with left brachiocephalic AVF, where thrombosis had spread to the left subclavian and internal jugular vein. Two patients had thrombosis extending through the basilic vein to within less than 3 cm of the axillary vein.

All patients were treated with local cooling, elevation of the arm, and analgesics. Antibiotic therapy was initiated in 20 (40%) patients due to elevated CRP and possible concomitant cellulitis. Anticoagulation therapy with low-molecular-weight-heparin, warfarin, fondaparinux, or rivaroxaban was initiated in 15 (30%) patients, while two patients (4%) received antiplatelet therapy. Six patients (12%) were hospitalized and extirpation of the AVF was performed after resolution of inflammation in 21 (42%) cases.

## Discussion

Our national cohort study of kidney transplant recipients with a functioning AVF at the time of transplantation provides new insight on the fate of AVFs after kidney transplantation. Kidney graft survival in our cohort was good. Furthermore, we found that approximately half of AVFs remained functional for many years after transplantation. The relatively young age and low prevalence of diabetic kidney disease compared with a recent Collaborative Transplant Study [9], may have influenced kidney graft survival and vascular access outcomes.

Nearly one-third of transplant recipients had some use of their AVF either in the immediate posttransplant period or later, and 40% of patients with graft failure started hemodialysis with their original pretransplant AVF. On the other hand, within a median follow-up of approximately 5 years, some AVF-related complications occurred in nearly one-third of kidney transplant recipients, the most common being aneurysmal enlargement and painful thrombosis with thrombophlebitis.

Looking at our data, AVFs after kidney transplantation were mainly used as vascular access for hemodialysis, usually at the beginning or end of graft life. It is known that approximately 2025% of deceased donor transplant recipients experience posttransplant DGF and need one or more hemodialysis sessions. Since the majority of these patients already have an existing vascular access [10], the placement of a dialysis catheter is not necessary. Unfortunately, kidney graft longevity is also limited. Due to the high likelihood that even successfully transplanted patients will eventually need to return to dialysis, some authors do not recommend ligation of a functional AVF after transplantation [11]. Therefore, the proper care of existing functional vascular access among kidney transplant recipients is crucial [12].

In our cohort, 40% of patients with graft failure were able to use their original pretransplant AVF, while in only 7% it had previously been ligated/extirpated. Moreover, at the time of graft failure, when some patients are undergoing intensive immunosuppressive therapy, the creation of a new vascular access might be associated with an increased risk of surgical and infectious complications. A preexisting functional AVF may therefore provide additional benefit for these patients. An AVF after kidney transplantation may also be used as a vascular access for plasma exchange or immunoadsorption in the treatment of posttransplant complications, such as antibody-mediated rejection or recurrence of primary focal segmental glomerulosclerosis. Some transplanted patients have low-quality veins or even problems with the central veins. An existing AVF may even solve the problem of vascular access for intravenous therapy, especially if the use of large veins is necessary.

The natural history of AVFs after kidney transplantation provides a fairly long fistula life in the majority of patients. In our cohort, 18% of AVFs stopped functioning in the first year after transplantation, 39% at 5 years and median AVF patency was almost 8 years after kidney transplantation. The main reason for AVF failure was spontaneous thrombosis, with a 5-year AVF patency of 61%, similar to 31% thrombosis and 7.5% ligation rate within 5.8 years of follow-up in a study by Patard et al. [13]. Thrombosed AVFs were forearm fistulas or PTFE grafts in which spontaneous thrombosis is likely to occur, due to a low or decreasing blood flow rate. Fistula dysfunction preceding definitive thrombosis is usually detected during routine dialysis procedures, so the lack of routine dialysis in transplanted patients may also be the reason for more frequent AVF thrombosis after successful kidney transplantation.

As our data show, AVF thrombosis in kidney transplant recipients may be associated with a high degree of local inflammation and thrombophlebitis. This is in contrast to the hemodialysis population, where this phenomenon is very rare. Severe thrombophlebitis can also occur after surgical AVF ligation. Lomonte et al. [14] reported on 11 kidney transplant and 8 hemodialysis patients who underwent AVF ligation. Painful thrombosis accompanied by edema and thrombophlebitis was the most common AVF-related complication in kidney transplant recipients, but not in hemodialysis patients. The main difference between the groups was immunosuppressive therapy in the transplant group. This is consistent with our data, although it is somewhat surprising that there is a stronger inflammatory response while the patient is on immunosuppressive therapy. The inflammation accompanying thrombosis may also be due to a higher thrombotic mass in enlarged tortuous aneurismal fistulas causing more severe thrombophlebitis.

There are no treatment guidelines for the management of AVF thrombosis after kidney transplantation. At our centre, after consultation with specialists in anticoagulant treatment, we usually treat AVF thrombosis with coexisting thrombophlebitis with fondaparinux, lowmolecular-weight-heparin, or rivaroxaban for 6 weeks, as with superficial thrombophlebitis [15]. More severe cases require treatment with antibiotics, analgesics and, in the case of expanding thrombosis, therapeutic anticoagulation.

The occurrence of complications related to vascular access is a common event in any dialysis unit [16, 17], but problems are not uncommon in the transplant population either [8]. Transplant nephrologists tend to focus on the management of immunosuppressive therapy, prevention of rejection, and prolongation of graft life, but occasionally forget about the management of vascular access, if there is one present [18]. In addition to thrombosis and thrombophlebitis, aneurysmal enlargement, high-flow fistulas, arm edema, steal syndrome, and trauma were complications associated with AVF. In published literature, the rate of aneurysm development varies widely in the dialysis population [19, 20], but there are no data on aneurysms in a functioning AVF after transplantation. Many AVF aneurysms after kidney transplantation are asymptomatic and problematic only for cosmetic reasons. These asymptomatic aneurysms can be safely observed [21], but if not monitored regularly, they can become large. There are some cases of “forgotten” aneurysmal fistulas reported in literature, which can lead to catastrophic bleeding after trauma or rupture [22]. Recently, a study showed that AVF aneurysms in patients undergoing immunosuppressive therapy were significantly larger and had a more intense Tlymphocytic infiltrate, suggesting that immunosuppressive therapy could play a role in aneurysm growth [23].

Our approach to AVF management after kidney transplantation was a conservative one. Surgery for AVF was performed only in patients with severe problems. The majority of AVF ligations or extirpations were performed because of aneurysms and high-flow AVFs. Recently, Fraser et al. [24] investigated the indications for and safety of AVF removal after kidney transplantation in 36 patients. The indications for AVF excision were aneurysm, pain, steal syndrome, thrombosis, heart failure, and venous hypertension. Only two patients (5%) experienced postoperative complications such as hematoma and wound infection. According to a recent retrospective American study, ligation of the AVF after transplantation is uncommon and generally reserved for patients with steal syndrome, AVF infection, or aneurysmal complications [25]. In a randomized controlled trial, prophylactic ligation of high-flow AVF (flow > 1500 ml/min) prevented high-output heart failure [26]. Based on these new data, the authors concluded that a more liberal approach to the closure of AVF after transplantation may be warranted [26]. Although a flow-reduction surgery might be even more appropriate and more conservative approach. Letachowicz et al. recently studied vascular access function and perspective in the kidney transplantation population. They reported that in the majority of transplant patients, vascular access blood flow was below the threshold of adverse cardiovascular effects of vascular access. Patients with high - flow AVFs (more than 1500 ml/min) were younger and had more proximally located AVFs. Less than 10% of patients had very limited options for future vascular access [27]. They also found that the proportion of patients with heart failure and dyspnea was higher in patients with proximally located AVFs (24% vs. 12%, *p* = 0.048) [28].

Little is also known about the attitude of kidney transplant recipients toward functioning AVF. Bardowska et al. investigated patients’ opinions on AVF ligation after successful kidney transplantation. In their cohort, 22.9% of recipients considered AVF closure, mainly for cosmetic reasons, and concerns about cardiac health. 38.5% of patients never wanted to ligate the AVF, and 38.5% of patients had no clear opinion. Paradoxically, patients with the worst kidney graft function and a distally located AVF had the highest proportion of individuals willing to ligate their vascular access [29].

As a whole, the current management of a functioning AVF in kidney transplant recipients remains controversial and does not rely on strong evidence-based data. A multicenter survey showed a considerable disagreement regarding AVF management among experts. Furthermore, routine vascular access surveillance was reported in only 29% of the responders [30]. Wilmink et al. have recently proposed guidelines regarding AVF closure after kidney transplantation, basing the decision on a trade-off between the estimated probability of future graft failure and the probability of future harm from a well-functioning fistula [31]. The individual risk of graft dysfunction and a return to chronic HD needs to be balanced [32]. There is also a clear need to educate patients about post-transplant AVF management options [29].

The strength of our study is the size of our national cohort, which includes more than 600 kidney transplant patients with a functioning AVF. Slovenian kidney transplant patients are all cared for and treated at a single national transplant centre, so the possibility of patient and data loss is low. In addition, our transplant centre is located next to a large dialysis centre employing nephrologists who are also dialysis vascular access surgeons and are keen to help with AVF problems. There are also some limitations worth mentioning. First, our study is observational and retrospective in nature. Second, we do not provide data on specific characteristics that might influence AVF patency and complications, such as diabetes, atherosclerosis, medications, AVF flow, central venous stenosis, and smoking.

## Conclusion

In conclusion, this study provides further information on the patency, utility and complications of AVFs in a national cohort of kidney transplant recipients. This information can be used to better discuss options for vascular access management in kidney transplant patients. Despite the possibility that routine AVF closure after kidney transplantation may reduce the risk of complications and be beneficial for cardiac function, it is important to recognize that a functional AVF can often be of use and that AVF closure reduces the possibility of further accesses for the patient. Our approach to AVF closure is therefore more conservative. We would advocate that an AVF should be ligated in the case of a high-flow fistula with evidence of impaired cardiac function and stable graft function, but should be preserved particularly in patients with significant graft dysfunction or chronic rejection, and especially in all patients with poor options for a new access. Vascular mapping to assess other AVF options in future should be mandatory before considering AVF closure. The decision to close an AVF after transplantation should always be made on an individual basis, taking into account AVF flow, potential complications, cardiac and kidney graft function, and ultimately the patient’s wishes.

## Data Availability

The datasets used and/or analysed during the current study are available from the corresponding author on request.
